# Supplementary material for: Discrimination of CpG Methylation Status and Nucleotide Differences in Tissue Specimen DNA by Oligoribonucleotide Interference-PCR
Source: Int J Mol Sci. 2020 Jul 20;21(14):5119. doi: 10.3390/ijms21145119 (PMC7404293; doi:10.3390/ijms21145119)
Supplement: Supplementary file 1 [file ijms-21-05119-s001.pdf]

Table S1. ORNs and primers used in this study.

Types	Names	Sequences (5' to 3')	Experiments	Number
ORNs	rGST-ORN3	ccuacaugaagagcagccgcua	Fig. 2 and Supplementary Fig. S2 (ORN_GA3)	-
	rGST-ORN2	uacaugaagagcagccgcua	Fig. 4 and Supplementary Fig. S1 (ORN_GA2)	-
	ORN_EGFR_L858	caguuuggccagcccaaaauc	Fig. 3 and Supplementary Fig. S3	-
	ORN_hCDKN2A-Bisul-Nest(U)-F (27483)	guggggaguaguauggaguuuuu	Figs. 6 and 7 and Supplementary Fig. S6 (ORN_p16_U)	-
	ORN_hCDKN2A-Bisul-Nest(M)-F (27481)	cggggaguaguauggaguuuuc	Figs. 6 and 7 and Supplementary Fig. S6 (ORN_p16_M)	-
Primers	rGST-M1-FP3	CCAGCATGATTCCAGGACAAAAG	Figs. 2 and 4, and Supplementary Figs. S1 and S2	28537
	rGST-M1-RP3	GGGGAGAATGAAGACTATGTGGG	Figs. 2 and 4, and Supplementary Figs. S1 and S2	28538
	rGST-M1-FP6	CACGAATCCTGTCACCAGTAAAA	Figs. 2 and 4, and Supplementary Figs. S1 and S2	28539
	rGST-M1-RP5	ATCTGAGGAAGAAGACAACCCAAG	Figs. 2 and 4, and Supplementary Figs. S1 and S2	28540
	hEGFR-Exon21-F4	TCTGTTTCAGGGCATGAACTACT	Fig. 3 and Supplementary Fig. S3	28556
	hEGFR-Exon21-R2	CACCCAGAATGTCTGGAGAGC	Fig. 3 and Supplementary Fig. S3	28287
	hGAPDH-0.3kbp-F	TGCCTCTTGTCTCTTAGATTTGG	Fig. 3	28554
	hGAPDH-0.3kbp-R	ATGGGATTTCCATTGATGACA	Fig. 3	28555
	hCDKN2A-Bisul-CpG-free-F	TTTTTAGAGGATTTGAGGGATAGG	Figs. 6 and 7, and Supplementary Figs. S4 and S6	27677
	hCDKN2A-Bisul-CpG-free-R	CTACCTAATTCCAATTCCCCTACAAACTTC	Figs. 6 and 7, and Supplementary Figs. S4 and S6	27678
	hACTIN-Bisul-F	TATGGGTTAGAAGGATTTTTATGTG	Fig. 7	28405
	hACTIN-Bisul-R	TTTACACCAACCTCATAACCTTATC	Fig. 7	28406

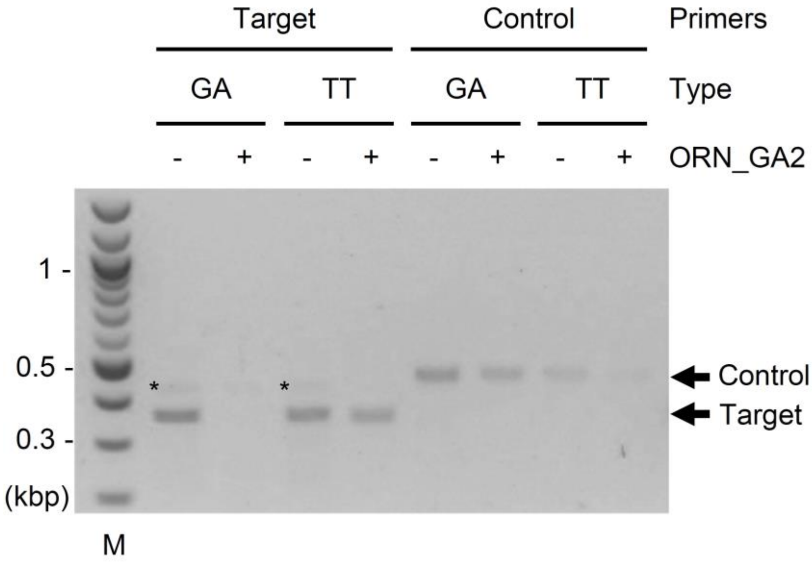

Figure S1. Results of ORNi-PCR using DNA extracted from AFPE rat liver specimens to discriminate a polymorphism of the *Gstm1* gene. ORNi-PCR with ORN_GA2 was performed as shown in Figure 2B. M, molecular weight marker. *, non-specific amplicons.

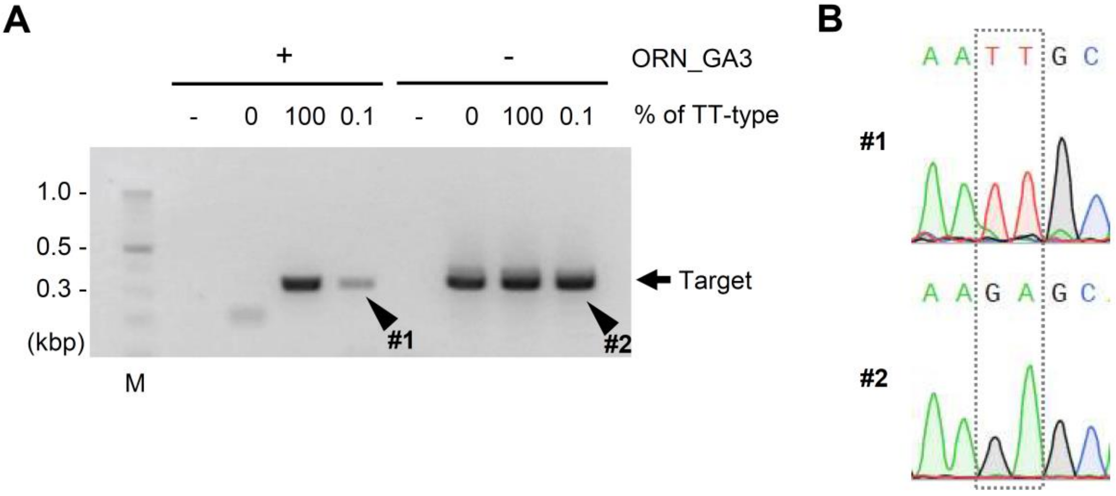

Figure S2. ORNi-PCR using DNA extracted from frozen rat liver specimens to discriminate a polymorphism in the *Gstm1* gene. (**A**) Results of ORNi-PCR. In this regard, 20 ng of DNA including GA-type *Gstm1* was mixed with DNA including TT-type *Gstm1* so that the TT-type *Gstm1* accounted for 0.1% of the total *Gstm1*. The mixed DNA was used for ORNi-PCR. ORNi-PCR was performed as shown in Figure 2B with 40 cycles of denaturation and annealing/elongation (60°C) steps. M, molecular weight marker. (**B**) Results of DNA sequencing analysis. ORNi-PCR and PCR amplicons shown in (A) were subjected to DNA sequencing analysis. Sequencing signals around the polymorphisms (Figure 2A) are shown.

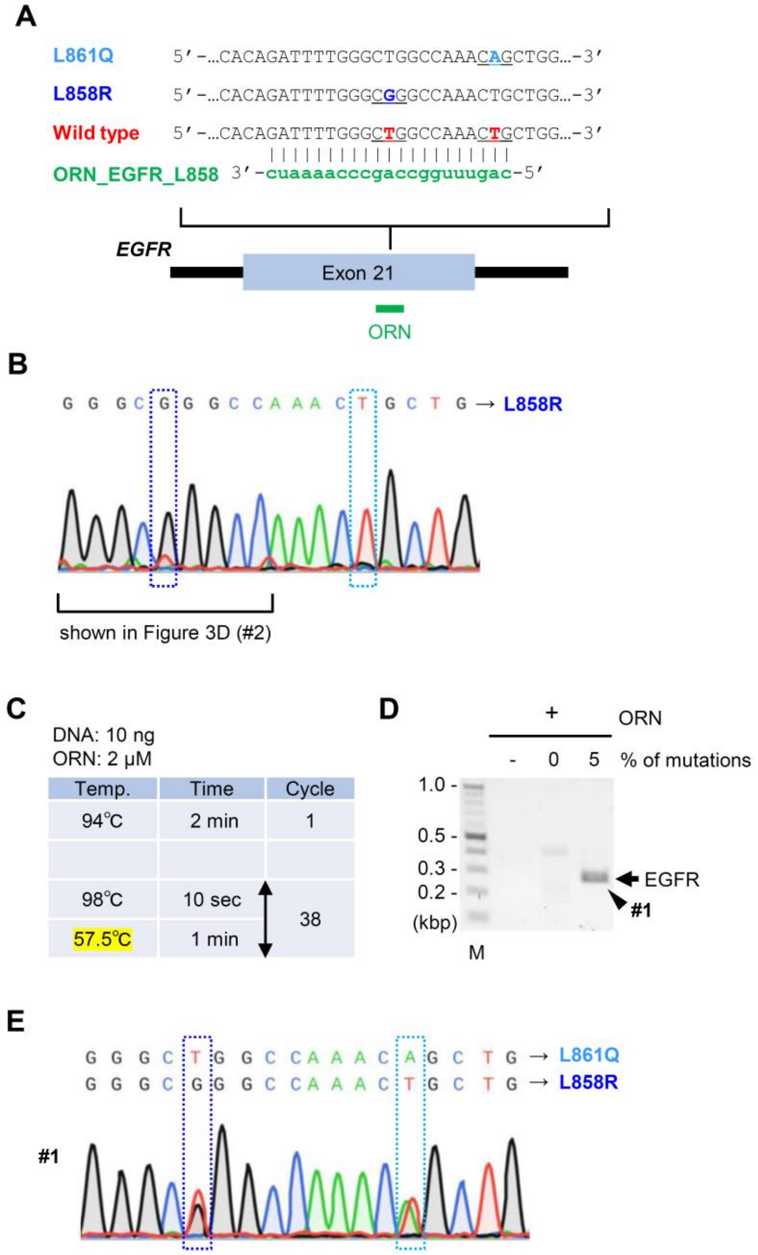

Figure S3. ORNi-PCR using DNA extracted from FFPE human specimens. (**A**) The position of ORN_EGFR_L858, which can also target a mutation corresponding to EGFR L861Q in the human *EGFR* gene. (**B**) The result of DNA sequencing analysis shown in Figure 3D (#2). Sequencing signals around the mutation corresponding to EGFR L861Q are also shown. (**C**) Experimental conditions for ORNi-PCR. (**D**) Results of ORNi-PCR. M, molecular weight marker. (**E**) Results of DNA sequencing analysis. An ORNi-PCR amplicon shown in (D) was subjected to DNA sequencing analysis. Sequencing signals around the mutations are shown.

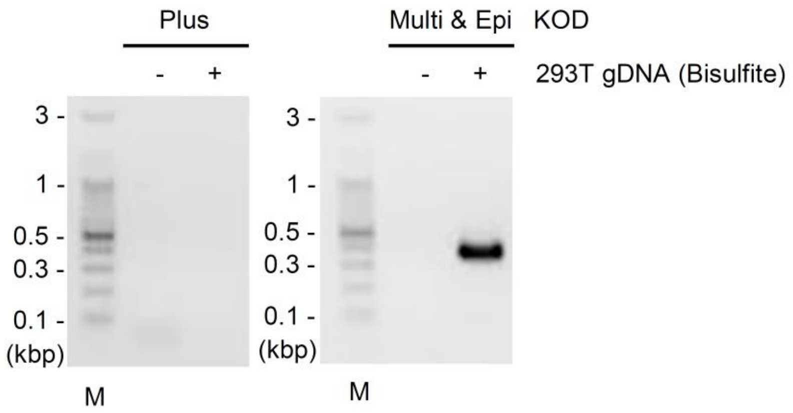

Figure S4. Comparison of two KOD DNA polymerases. KOD -Plus- Ver. 2 and KOD -Multi & Epi-™ were employed for ORNi-PCR using bisulfite-treated gDNA.

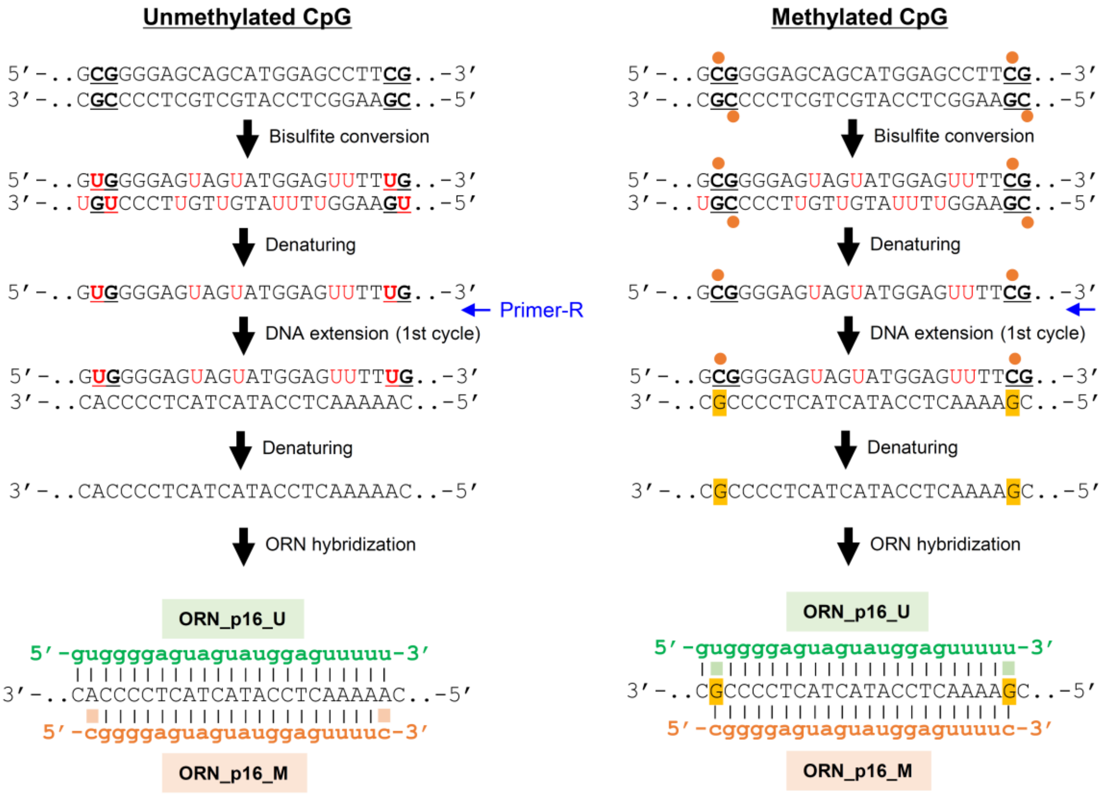

Figure S5. Schematic diagram of bisulfite treatment followed by ORNi-PCR with ORN_p16_U or ORN_p16_M.

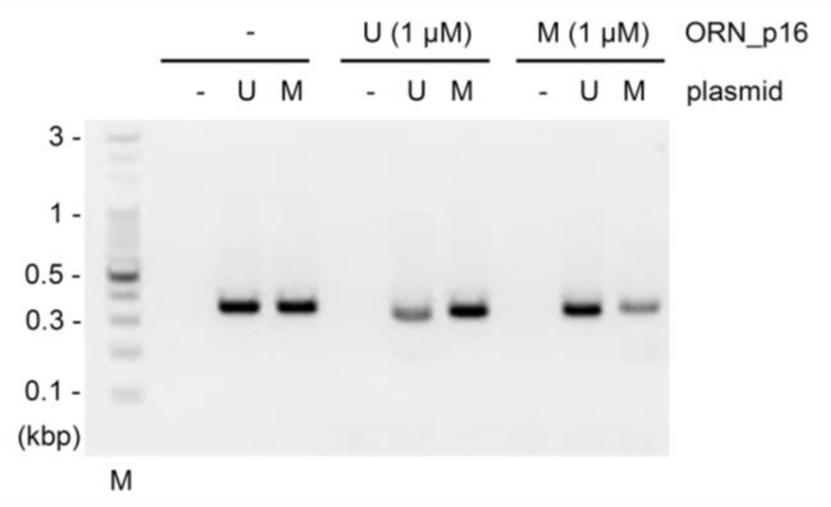

Figure S6. Results of ORNi-PCR. ORNi-PCR with 1 µM of each ORN was performed as shown in Figure 6D.
